# Hes4 Controls Proliferative Properties of Neural Stem Cells During Retinal Ontogenesis

**DOI:** 10.1002/stem.1231

**Published:** 2012-09-11

**Authors:** Warif El Yakoubi, Caroline Borday, Johanna Hamdache, Karine Parain, Hong Thi Tran, Kris Vleminckx, Muriel Perron, Morgane Locker

**Affiliations:** a UPR CNRS 3294, University Paris-SudOrsay, France; b Department of Molecular Biomedical Research, VIB Ghent UniversityGhent, Belgium; c Department of Biomedical Molecular Biology, Ghent UniversityGhent, Belgium

**Keywords:** Retina, Neural stem cells, *Hes4/XHairy2*, Wnt and Hedgehog signaling, Cell cycle kinetics

## Abstract

The retina of fish and amphibian contains genuine neural stem cells located at the most peripheral edge of the ciliary marginal zone (CMZ). However, their cell-of-origin as well as the mechanisms that sustain their maintenance during development are presently unknown. We identified *Hes4* (previously named *XHairy2*), a gene encoding a bHLH-O transcriptional repressor, as a stem cell-specific marker of the *Xenopus* CMZ that is positively regulated by the canonical Wnt pathway and negatively by Hedgehog signaling. We found that during retinogenesis, *Hes4* labels a small territory, located first at the pigmented epithelium (RPE)/neural retina (NR) border and later in the retinal margin, that likely gives rise to adult retinal stem cells. We next addressed whether Hes4 might impart this cell subpopulation with retinal stem cell features: inhibited RPE or NR differentiation programs, continuous proliferation, and slow cell cycle speed. We could indeed show that *Hes4* overexpression cell autonomously prevents retinal precursor cells from commitment toward retinal fates and maintains them in a proliferative state. Besides, our data highlight for the first time that Hes4 may also constitute a crucial regulator of cell cycle kinetics. *Hes4* gain of function indeed significantly slows down cell division, mainly through the lengthening of G1 phase. As a whole, we propose that Hes4 maintains particular stemness features in a cellular cohort dedicated to constitute the adult retinal stem cell pool, by keeping it in an undifferentiated and slowly proliferative state along embryonic retinogenesis. Stem Cells 2012;30:2784–2795

## INTRODUCTION

Contrasting with the mammalian situation, the retina of adult fish and amphibians contains a population of neural stem cells, which allow continuous tissue growth throughout the animal life as well as regeneration following retinal damage [1]. These retinal stem cells (RSCs) reside in a permanently proliferating region located at the margin of the retina, known as the ciliary marginal zone (CMZ; [2]). The spatial organization of the CMZ mirrors the temporal sequence of retinal development with stem cells being found in its most peripheral part, successively followed more centrally by actively dividing progenitors and then by their postmitotic progeny [3, 4].

Much progress has been made these past few years in the characterization of CMZ neural stem cells. This includes the formal demonstration of their multipotency and self renewal ability [5, 6], advance in the description of their niche [7, 8] and the identification of candidate genes and signaling pathways to regulate their postembryonic activity [9–17]. However, several questions remain unresolved and in particular, that of their embryonic origin. Whether they arise from a discrete population of cells and how they escape from cell cycle exit and differentiation signals during retinal development is hitherto unknown. Comprehensive analysis of RSC ontogeny and properties necessitates reliable markers to formally identify these immature cells within proliferating heterogeneous cell populations *in vivo*. To gain insight into the molecular signature of RSCs, we recently performed a large-scale expression screen in the *Xenopus* CMZ [14]. Among identified RSC markers, we retrieved the *Hes4* gene (previously known as *XHairy2* in *Xenopus* and ortholog of zebrafish *Her9* and chick *cHairy1*) that encodes a transcriptional repressor of the bHLH-O family.

*Hes* family genes are well known as Notch transcriptional targets that can regulate cellular differentiation, cell fate decisions, and embryonic patterning in various developmental systems [18, 19]. Notably, several members of this family, including *Hes1*, which is closely related to *Hes4* in terms of sequence similarity [18], have been intensively studied in the developing vertebrate brain. In this context, several lines of evidence converge toward a role in boundary formation and maintenance of neural stem/progenitor cells, mainly through prevention of neuronal differentiation [20–27]. *Hes1* is expressed as well in the embryonic retina, where it regulates distinct aspects of eye morphogenesis and is required for proper timing of neurogenesis [28–30]. *Hes1* also emerged as a safeguard of cellular quiescence, through protection against terminal differentiation and permanent cell cycle arrest [31, 32]. In contrast to *Hes1*, the *Hes4* gene was largely ignored in mammalian studies, presumably due to the absence of an ortholog in mouse. It is, however, expressed in humans and has been shown to be involved in several aspects of other vertebrate species development. In particular, Hes4 proved to play a significant role in maintaining the undifferentiated state of *Xenopus* neural crest cells [33–35] and zebrafish inner ear progenitors [36]. In addition, a recent publication by Kubo and Nakagawa identified the chick *Hes4* ortholog, *cHairy1* [37], as highly expressed in the CMZ and required for the maintenance of this structure downstream Wnt signaling [38]. We thus decided to gain further insights into *Hes4* expression and function in the developing *Xenopus* retina.

We found that in contrast to mouse *Hes1* [28, 30] and chick *cHairy1* [39], *Hes4* is not expressed in the neural retina (NR) at any stage examined but labels the presumptive retinal pigmented epithelium (RPE) and forming CMZ before being restricted to stem cells of the mature retina. Wnt and Hedgehog signaling pathways contribute to this dynamic expression pattern through positive and negative regulation, respectively. Finally, functional analysis revealed that Hes4 imparts retinal cells with stem-like properties: inhibited commitment toward RPE and neuronal fate, prolonged proliferative capacities, and slow cell cycle kinetics.

## MATERIALS AND METHODS

### Embryo Collection, Transgenic Line

*Xenopus laevis* embryos were obtained by conventional methods of *in vitro* fertilization. *Xenopus tropicalis* transgenic animals carrying the Wnt reporter pbin8LefdGFP construct have previously been described [40] and were obtained by natural fertilization between a wild-type female and a transgenic male carrying a single insertion of the transgene [41]. All experiments were approved by the Direction Départementale des Services Vétérinaires de l'Essonne, Evry, France.

### Pharmacological Treatments

Cyclopamine (20 μM; LC Laboratories, Woburn, MA, http://lclabs.com), purmorphamine (100 μM; Calbiochem, San Diego, CA, http://www.emdbiosciences.com), DAPT (N– [N–(3,5–Difluorophenacetyl)-L-alanyl]-S-phenylglycine t-butyl ester; 100 μM; Sigma, St Louis, MO, http://www.sigma aldrich.com), and IWR1 (Inhibitor of Wnt Response 1; 50 μM; Sigma) were applied to the embryo culture medium from stage 25 to stage 35. BIO (6-bromoindirubin-3′-oxime; 20 μM; Sigma) was applied for 1 hour on stage 25 embryos, which were then rinsed and kept in drug-free medium for 15 or 24 hours [41]. Effectiveness of drug treatment was systematically assessed through whole mount *in situ* hybridization by checking the expression of known target genes of the considered pathway: *Gli1* or *Patched1* (*Ptc1*) for Hedgehog [12], *CyclinD1* for Wnt [10], and *HRT1* for Notch [42].

### Expression Constructs

*pCS2-Flag-Hes4* (previously named *XHairy2*), *pCS2-Hes4-myc-GR* (inducible construct fused to the dexamethasone-responsive hormone-binding domain of the human glucocorticoid receptor (GR); referred as *Hes4-GR* in the text [34]), *pCS2-Hes2* [43], pCS2-X*gadd-45γ* [44], *pCS2-GFP* (a gift from David Turner), and *pCS2-LacZ* (a gift from Nancy Papalopulu) were previously described. *pCS2-Hes4-myc-VP16-GR* encodes a Hes4 glucocorticoid-inducible antimorphic variant where the VP16 transactivation domain is fused to the carboxylterminus of Hes4. It was generated by subcloning the *Hes4* coding sequence into a *pCS2-myc-VP16-GR* vector after polymerase chain reaction (PCR) amplification. Details on the cloning procedure are available upon request.

### Microinjections and *In Vivo* DNA Lipofection

*Xenopus laevis Hes4* gene exists as two alloalleles, *Hes4a* and *Hes4b*, and the later was used in all overexpression experiments. Capped sense RNAs were transcribed using the mMessage mMachine SP6 kit (Ambion, Austin, TX, http://www.ambion.com). 500 pg of mRNA was injected animally into one or two blastomeres at the two-cell stage, together with *GFP* or β-*galactosidase* mRNA (250 pg). Protein activity of GR chimeric constructs was induced by incubating the embryos in 4 μg/ml dexamethasone (Sigma) from stage 12. Treating embryos before this stage lead to severe developmental defects and frequently to developmental arrest. Morpholino oligonucleotides against both alloaleles (“*Hes4* Mo”; [34]) were injected into one blastomere at the four- or eight-cell stage (8–20 ng). Lipofection experiments were performed at stage 17/18 as previously described [45].

### *In Situ* Hybridization

Digoxigenin-labeled antisense RNA probe synthesis and whole-mount *in situ* hybridization were performed as previously described [14]. Embryos were then vibratome sectioned (50 μM). Following image capture, labeling area was manually delineated and quantified (in Pixel^2^) in both the dorsal and ventral CMZ using Adobe Photoshop CS4 software. Shown in graphs are percentage of staining area increase/decrease compared to the control.

### BrdU/EdU Incorporation and Immunohistochemistry

Embryos were injected intra-abdominally with BrdU (5-bromo-2′-deoxyuridine; 10 mM; Sigma) or EdU (5-ethynyl-2′-deoxyuridine; 1 mM; Invitrogen, Carlsbad, CA, http://www. invitrogen.com) for various durations, depending on the experiment. For birthdating analyses, embryos were injected every 10–12 hours from stage 34 to stage 41. Immunohistochemistry was performed on 12 μm-cryostat or -paraffin sections as previously described [12, 41]. Antibodies used are listed in Supporting Information Table 1. Cell nuclei were counterstained with Hoechst (Sigma). Detection of EdU-labeled cells was carried out with the Click-iT EdU Imaging Kit (Molecular Probes, Eugene, OR, http://probes.invitrogen.com). Fluorescent staining was visualized with a M2 Zeiss microscope. Images were captured using a digital Axiocam MRc camera and processed with AxioVision REL 7.8 and Adobe Photoshop CS4 softwares. Retinal area was manually delineated on transverse sections and quantified (in Pixel^2^) using Adobe Photoshop CS4 software.

### Analysis of Cell-Cycle Parameters

Growth fraction (GF; proportion of proliferative cells), total cell cycle length (*T*_C_), and S-phase length (*T*_S_) were determined following EdU cumulative labeling using the Excel sheet provided by Dr R. Nowakowski [46]. Mitotic index and percentages of EdU-labeled mitosis were measured as previously described [12]. The time required for half-maximal appearance of EdU labeling in the mitotic population was taken as an estimation of the average G2 length (*T*_G2_; [47]). M-phase duration (*T*_M_) was calculated after determining the proportion of cells that were in mitosis (% M), taking into account *T*_C_ and GF, as determined by cumulative EdU labeling: *T*_M_ = % M × GF × *T*_C_. Finally, G1 duration (*T*_G1_) was deduced from the above values as *T*_G1_ = *T*_C_ − (*T*_S_ + *T*_G2_ + *T*_M_).

### Reverse Transcription and Quantitative Real Time PCR

Reverse transcription of retinal mRNA and quantitative real time PCR (qPCR) were performed as described previously [41]. PCR primer sequences are listed in Supporting Information Table 2.

### Quantification and Statistical Analysis

All experiments were performed at least in duplicate. Shown in figures are results from one representative experiment. In each histogram, values are given as mean ± SEM. In experiments involving two conditions, statistical analysis was performed by Student's *t* test, while in those involving the comparison of more than two treatments one-way ANOVA followed by Tukey's post hoc tests were performed (NS: not significant; *, *p* < .05; **, *p* < .01; ***, *p* < .001).

## RESULTS

### *Hes4* Expression Likely Reveals the Embryonic Cell-of-Origin of Adult RSCs

**Figure 1:**
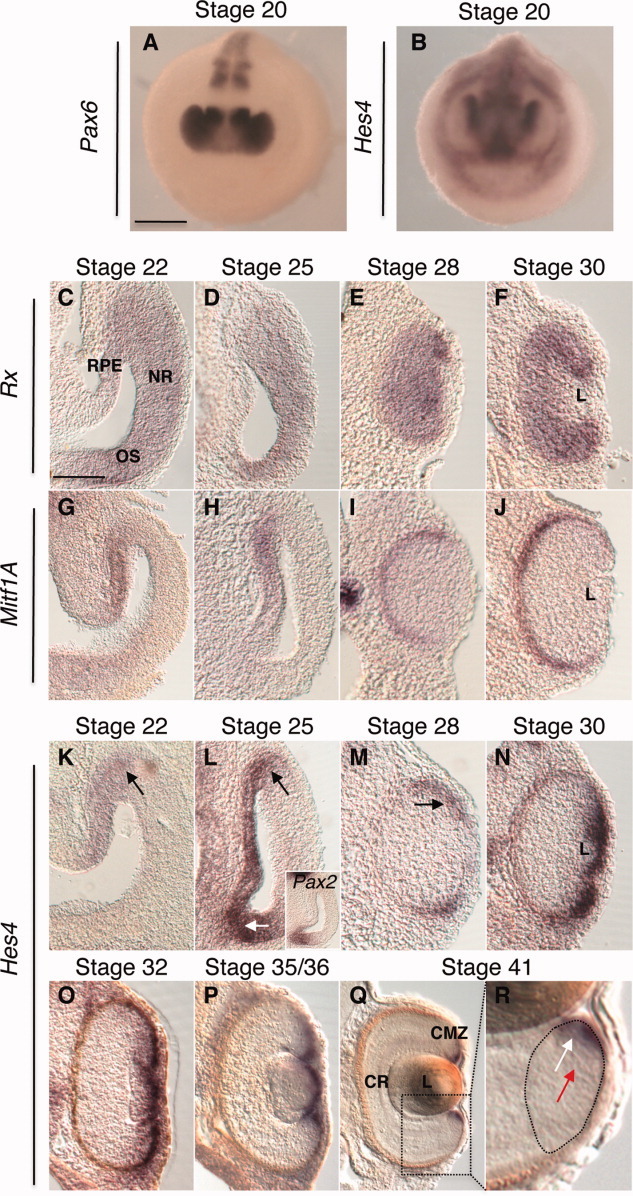
*Hes4* expression during retinogenesis. Comparative *in situ* hybridization analysis of *Hes4* expression profile **(B, K–R)** with that of *Pax6***(A)**, *Rx***(C–F)***Mitf1A***(G–J)**, and *Pax2* (inset in **L**). White arrow in **(L)** point to the OS. Black arrows in **(K–M)** indicate the RPE/NR border (K, L) and the retinal margin (M). **(Q)** corresponds to a magnification of the CMZ delineated in **(R)**. White and red arrows, respectively, indicate stem cell and progenitor zones of the CMZ. Scale bars = 300 μm (A, B) or 50 μm (C–R). Abbreviations: CMZ, ciliary marginal zone; CR, central retina; L: lens; NR, neural retina; OS, optic stalk; RPE, retinal pigmented epithelium.

The embryonic lineage that gives rise to adult RSCs in *Xenopus* is currently unknown. Gene expression profile within the CMZ has proved to mirror the temporal genetic sequence of retinal development [3, 4, 48]. As *Hes4* labels the most peripheral stem cell-containing part of the CMZ ([14] and Figure 1Q, 1R), we hypothesized that establishing its expression profile during retinogenesis might retrospectively give clues about RSC ontogenesis. We thus analyzed the distribution of its transcripts at key stages of eye development and compared it with that of optic field (*Pax6*), NR (*Rx*), optic stalk (OS; *Pax2*), and RPE (*Mitf1A*) markers (Fig. 1; Supporting Information Fig. S1). At late neurula stage, *Hes4* expression was observed in a restricted proximo-dorsal region of the optic field, clearly contrasting with that of *Pax6* (Fig. 1A, 1B). In line with this, analysis on retinal sections at the optic vesicle stage revealed that *Hes4* stained the *Mitf1A*-expressing presumptive RPE and was not detected in the *Rx*-positive retinal neuroepithelium, except in its most dorsal part at the border with the RPE (Fig. 1K, 1L). Of note, from stage 24–25, *Hes4* transcripts were additionally observed in the OS (coinciding with *Pax2* expression; Fig. 1L). During optic cup formation, *Hes4* expression progressively declined in the differentiating central RPE while remaining high in its peripheral part and in the NR margins (Fig. 1M–1O). Staining in the margin progressively superimposed with the forming CMZ to finally get restricted to its most peripheral region (Fig. 1P–1R). As a whole, *Hes4* expression delineates a territory clearly distinct from both the differentiating NR and RPE, that is first located in the dorsal part of the optic vesicle, then at the peripheral margin of the optic cup, and that finally coincides with RSC location following completion of embryonic retinogenesis. Importantly, such a dynamic expression pattern strongly resembles that of *Gli3* and *Smo*, two previously described stem-cell specific markers [49]. In addition, we found that the same held true for two other genes that we recently identified as being expressed at the extreme tip of the CMZ, namely *Id2* and *Wnt8b* (Supporting Information Fig. S2 and [41]). Altogether, these data thus suggest that adult RSCs originate from a discrete population of cells located at the RPE/NR border of the optic vesicle.

### *Hes4* Expression Is Positively Regulated by Wnt Signaling and Negatively by the Hedgehog Pathway

**Figure 2:**
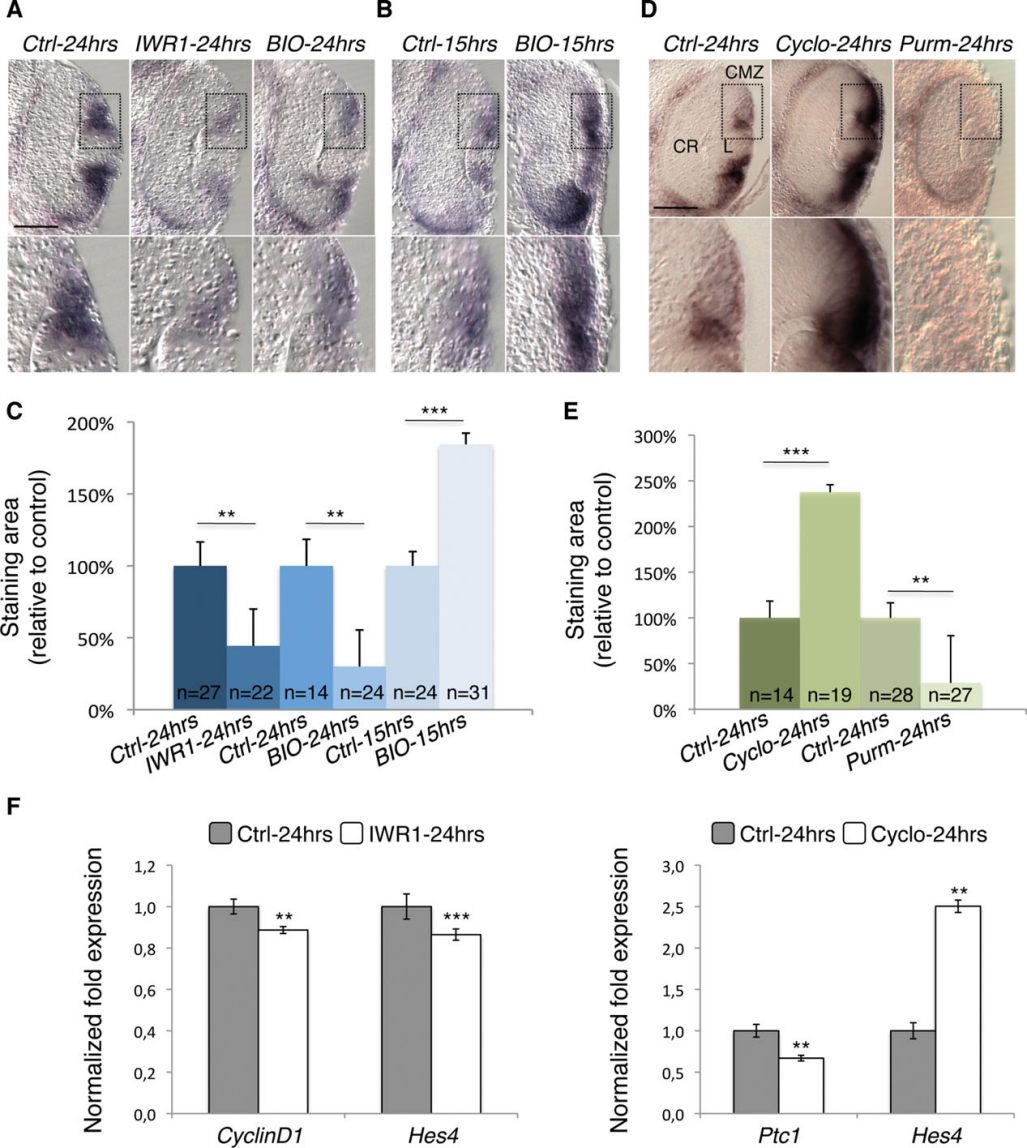
Wnt and Hedgehog signalings affect *Hes4* expression in an opposite manner. **(A–E):***In situ* hybridization analysis of *Hes4* expression on retinal sections, following a 24-hour (from stage 25 to stage 35; A, C–E) or a 15-hour (from stage 25 to stage 32; B, C) treatment with the indicated drug. Shown beneath each retinal section is a higher magnification of the dorsal CMZ delineated with dotted lines. (C, E): Quantification of *Hes4* staining area in the dorsal CMZ in each condition. **(F):** Quantitative polymerase chain reaction analysis of retinal *Hes4* expression at stage 35 following 24 hours of Hedgehog or Wnt signaling inhibition. *Ptc1* and *CyclinD1* serve as controls of drug efficiency. Scale bar = 50 μm. Abbreviations: BIO, 6-bromoindirubin-3′-oxime; CMZ, ciliary marginal zone; CR, central retina; Cyclo, cyclopamine; IWR1, inhibitor of wnt response 1; L, lens; N, number of analyzed sections; Purm, purmorphamine.

We then aimed at identifying the signaling pathways regulating *Hes4* embryonic expression. A potential candidate is the canonical Wnt pathway since it proved to be active in the peripheral retina of various species [10, 50, 51] and was shown to regulate CMZ expression of *cHairy1*, the *Hes4* ortholog in chick [38]. However, the status of Wnt activity during embryonic retinogenesis of *Xenopus* has never been investigated in detail. Taking advantage of a *Xenopus tropicalis* reporter line carrying a destabilized *eGFP* downstream a synthetic Wnt-responsive promoter [40], we thus analyzed the profile of Wnt activity in the developing retina. Except in the presumptive RPE and OS of the optic vesicle where it was not detected, *eGFP* staining strikingly superimposed with that of *Hes4* (Supporting Information Fig. S3). Consistent with a role in promoting *Hes4* expression, we found that pharmacological blockade of Wnt signaling from stage 25 to stage 35 (24-hour IWR1 treatment) significantly lowered *Hes4* levels in the CMZ, as inferred from both *in situ* hybridization and qPCR analyses (Fig. 2A, 2C, 2F). Unexpectedly, similar inhibition was observed upon a 24-hour treatment with BIO, an activator of the Wnt pathway (Fig. 2A, 2C). Shortening the treatment to 15 hours, however, led to a consistent increase of *Hes4* labeling (Fig. 2B, 2C). As Hes1 is known to repress its own expression [52], these results led us to investigate whether this might also be the case for Hes4. In line with this, we found that misexpression of the Hes4 antimorphic variant Hes4VP16-GR (which transforms the Hes4 repressor into a transcriptional activator) led to ectopic *Hes4* staining in the epidermis of injected embryos (Supporting Information Fig. S4A). Additionally, morpholino-mediated *Hes4* knockdown significantly enhanced *Hes4* expression in its endogenous domain at the neurula stage (Supporting Information Fig. S4B). As a whole, these results suggest that *Hes4* levels in the forming CMZ are positively regulated by the Wnt pathway and modulated by a negative feedback loop.

We next investigated the impact of Hedgehog signaling based on previous data in mouse retinal explants showing that *Hes1* expression is stabilized by the Shh effector Gli2 [53]. As a first attempt to address this issue, we compared *Hes4* expression pattern during retinogenesis with that of *Gli1* as a readout of Hedgehog signaling activity (Supporting Information Fig. S3). As previously described [12, 49], *Gli1* transcripts were detected at high levels in the presumptive RPE and OS of stage 25 embryos and at low levels in the NR and NR/RPE border. Strikingly, upon optic cup formation, *Gli1* and *Hes4* clearly exhibited complementary expression patterns, with *Gli1* being mainly detectable in the differentiating RPE and *Hes4* restricted to the peripheral margin of the retina. Finally, in the mature retina, *Gli1* labeling was detected in the central CMZ and was faint in its most peripheral *Hes4*-expressing part. These results suggest that in contrast to mouse *Hes1*, *Hes4* might be downregulated by Hedgehog signaling. In line with this, we found that Hedgehog pathway inhibition with cyclopamine significantly enhanced *Hes4* expression in the CMZ (Fig. 2D–2F). Conversely, Hedgehog activation with purmorphamine strongly reduced *Hes4* staining (Fig. 2D, 2E). Altogether, it is likely that Hedgehog-mediated inhibition of *Hes4* contributes to restrain its expression domain to the peripheral margin of the optic cup and to the stem cell compartment of the CMZ.

Although *Hes* genes are recognized as canonical targets of Notch signaling, a growing wealth of data indicate that they may function independently in several contexts, including the retina [38, 53]. Considering the above data, we investigated whether this might be the case for *Hes4* as well. We thus blocked the Notch pathway with DAPT, a chemical compound that inhibits the γ-secretase-dependent release of the Notch intracellular domain and was shown to repress *Hes1/5* expression in the chick retina [54]. Contrasting with the effects of Wnt and Hedgehog signaling perturbations in the same time window, *Hes4* levels were not significantly lowered by a 24-hour DAPT exposure (Supporting Information Fig. S5B, S5E, S5F) and were only affected when treatment duration was extended to 48 hours (Supporting Information Fig. S5D, S5E). Such a delay suggests that the Notch pathway does not directly contribute to *Hes4* regulation in the forming CMZ.

### *Hes4* Loss of Function Severely Impairs Eye Formation

**Figure 3:**
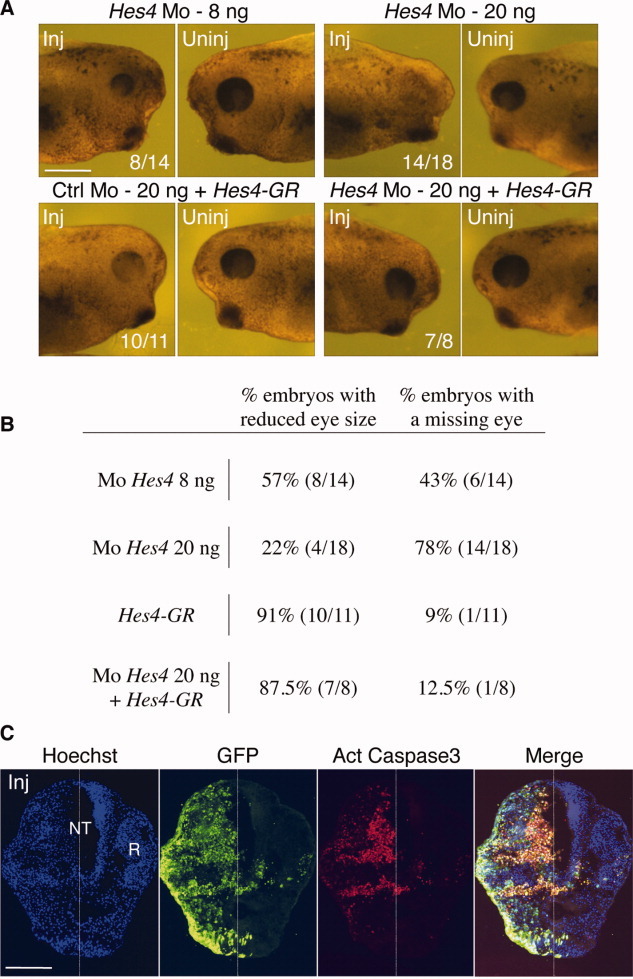
*Hes4* is required for proper eye formation. **(A, B):** Analysis of *Hes4* loss of function. *Hes4* or Ctrl Mo were injected at the indicated dose with or without *Hes4-GR* mRNA. **(A):** Typical pictures of stage 37 injected embryos (lateral view of the head). Note that embryos injected with *Hes4-GR* mRNA alone exhibit a defective RPE pigmentation and a slightly smaller eye. **(B):** Quantification of morphant and rescued embryos exhibiting the indicated phenotypes. **(C):** Transverse sections of a stage 25 morphant embryo (injected with 8 ng *Hes4* Mo) immunostained with anti-active caspase3 and anti-GFP (to visualize the injected side). Scale bars = 300 μm (A) or 100 μm (C). Abbreviations: GR, glucocorticoid receptor; GFP, green fluorescent protein; Inj, injected side; NT, neural tube; R, retina; Uninj, uninjected side.

We next aimed at addressing *Hes4* function during retinal development through a knockdown experiment. Morphological analysis of stage 37 tadpoles injected with *Hes4* Morpholinos (*Hes4* Mo) revealed severe dose-dependent eye defects ranging from a strong reduction of eye size to a complete absence of eye (Fig. 3A). No discernable eye malformations were observed upon injection of a standard Morpholino (Ctrl Mo; data not shown). In order to exclude a potential toxicity of the *Hes4* Mo, we tested whether the eye-absent morphant phenotype could be rescued upon coinjection of *Hes4* Mo with an inducible *Hes4* construct (*Hes4-GR*) devoid of *Hes4* Mo target sequence. The percentage of eye-absent embryos was scored in the different conditions, and we found that it dropped from 78% in *Hes4* Mo-injected embryos to 12.5% in *Hes4* Mo plus *Hes4-GR*-injected ones (Fig. 3B). Such a rescue clearly suggests that the loss of eye is a specific phenotype of *Hes4* knockdown. Consistent with this, severe eye defects were observed as well following overexpression in the presumptive neurectoderm of the antimorphic *Hes4* variant *Hes4VP16-GR* (data not shown).

Since two previous studies in *Xenopus* reported increased apoptosis at neurula stages following *Hes4* loss of function [33, 35], we wondered whether cell death might account as well for the observed eye malformations. We thus performed anti-active caspase3 immunostaining on stage 25 retinal sections from *Hes4* Mo injected embryos. Massive apoptosis was evident on the injected side (Fig. 3C). Thus, as previously proposed in neural crest stem cells [33, 35], Hes4 might be required for cell survival in the retina. Of note, cell death was not restricted to *Hes4*-expressing tissues and spread in particular to the NR, which might be a secondary consequence of the loss of surrounding tissues.

### *Hes4* Misexpression Prevents RPE Differentiation and Neuronal Commitment of Retinal Precursor Cells

**Figure 4:**
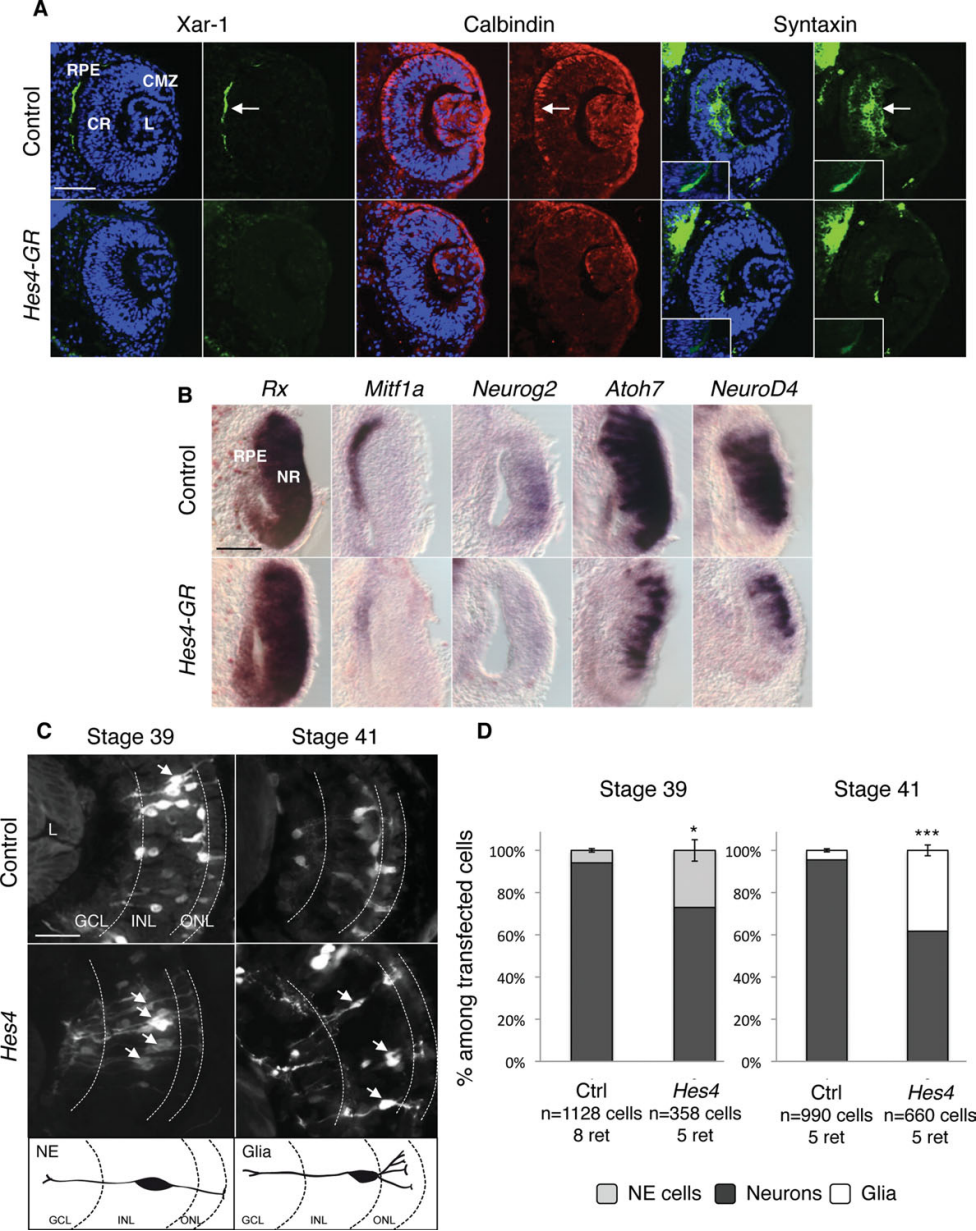
*Hes4* misexpression inhibits neuronal and RPE differentiation. **(A):** Immunofluorescence analysis of cell-type-specific marker expression in stage 37 retinas, following *Hes4-GR* mRNA injection. Arrows point to the staining in the RPE (Xar-1), photoreceptors (calbindin) or interneuron and ganglion cell fibers (syntaxin). The optic nerve is shown in insets. **(B):***In situ* hybridization analysis with the indicated probe in stage 25 retinas, following *Hes4-GR* mRNA injection. **(C, D):** Analysis of cell type distribution in stage 39 or 41 retinas, following *Hes4* lipofection. (C) Typical sections of retinas transfected with *GFP* alone (control) or *GFP* plus *Hes4*. Arrows indicate NE at stage 39 or Müller glial cells at stage 41. Respective morphologies of these cells are illustrated on the schematics below. (D) Quantification of NE, neurons, and glia among transfected cells at stage 39 or 41. Scale bars = 50 μm (A, B) or 25 μm (C). Abbreviations: CMZ, ciliary marginal zone; CR, central retina; GR, glucocorticoid receptor; GCL, ganglion cell layer; INL/ONL, inner/outer nuclear layer; L, lens; NE, neuroepithelial cell; NR, neural retina; RPE, retinal pigmented epithelium.

Since the dramatic phenotype of *Hes4* knockdown precludes any further functional investigation, we turned to a gain of function strategy using the *Hes4-GR* inducible construct. Morphological analysis at stage 37 revealed a defective RPE pigmentation (Fig. 3A) and a slight reduction of retinal size in *Hes4-GR*-injected embryos (23% ± 2.5% decrease of retinal surface compared to controls, as measured on 19 and 37 sections, respectively; *p* < .001). Effects on retinal cell type differentiation were then monitored using specific markers (Fig. 4A). In line with the aforementioned defects in retinal pigmentation, *Hes4* overexpressing retinas displayed a virtual absence of XAR-1 expression, a marker of the differentiated RPE. In addition, substantial perturbations in laminar organization were observed in the central retina (CR). In line with this, both rhodopsin (data not shown) and calbindin stainings were severely reduced suggesting that rods and cones were profoundly missing. Syntaxin expression was strongly decreased in the inner and outer plexiform layers as well as in the optic nerve, consistent with defective interneuron and ganglion cell production. Thus, *Hes4* misexpression drastically impairs both RPE and neuronal differentiation.

To get further insights into this phenotype, we checked the status of several markers at earlier stages by *in situ* hybridization (Fig. 4B). We observed that *Rx* expression was not affected suggesting that *Hes4*-overexpressing cells retain a retinal identity. However, *Mif1A* staining was strongly reduced compared to controls, consistent with an impairment of RPE determination. Besides, expression of the proneural genes *Neurog2*, *Atoh7*, and *NeuroD4* (previously called *XNgnr-1*, *Xath5*, and *Xath3*, respectively) was also decreased, suggesting that Hes4 inhibits neurogenesis by preventing commitment of precursor cells toward a neuronal fate. Consistent with this, we found that the antimorphic Hes4 variant Hes4VP16-GR was able to induce ectopic neurogenesis, as inferred by *N-tubulin* staining following mRNA injection in the presumptive epidermis (Supporting Information Fig. S4A).

We next followed the fate of *Hes4*-overexpressing cells in a clonal analysis using *in vivo* lipofection. At stage 39, most cells in control clones were already differentiated into neurons as judged by their position and morphology. In contrast, an important proportion of *Hes4*-overexpressing cells still exhibited a neuroepithelial morphology characteristic of undifferentiated neural precursors (Fig. 4C, 4D). Analysis at stage 41 revealed that this delayed differentiation eventually ended up with an excessive production of Müller glial cells at the expense of neurons (Fig. 4C, 4D). In addition to their typical morphology, Müller cell identity was further confirmed by immunostaining using an anti-CRALBP antibody (data not shown). These data indicate that *Hes4* overexpression cell autonomously delays differentiation and reduces neurogenesis. As a whole, these results suggest that *Hes4* maintains cells of the retinal margin in an undifferentiated state during embryonic retinogenesis.

### *Hes4* Misexpression Maintains Retinal Precursors in a Proliferative State

**Figure 5:**
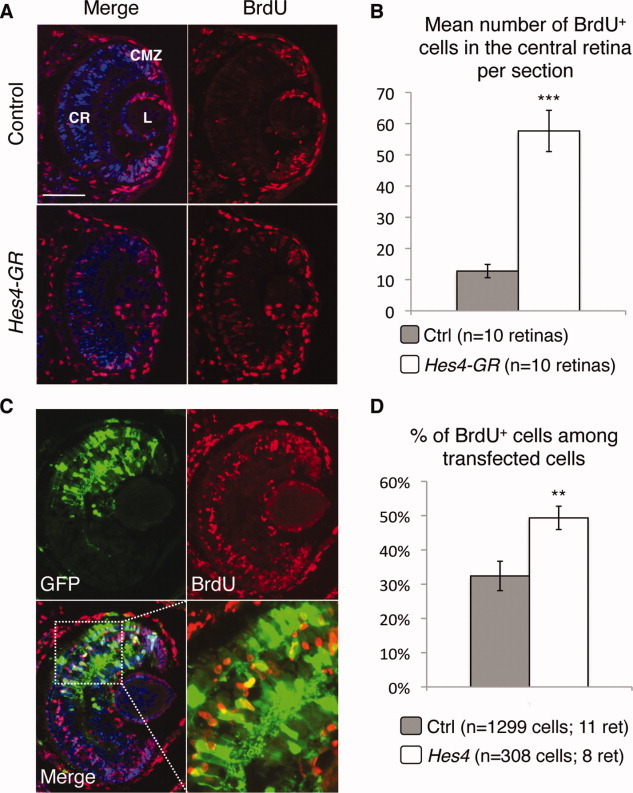
*Hes4* overexpression delays cell cycle exit. **(A, B):** BrdU incorporation assay (3-hour pulse) at stage 37, following *Hes4-GR* mRNA injection. (A): Typical retinal sections immunostained with anti-BrdU. (B): Corresponding quantification. **(C, D):** Birthdating experiments in *Hes4* lipofected retinas. (C): Typical retinal section immunostained for both GFP and BrdU, following continuous BrdU exposure from stage 34 to 41. (D): Percentage of BrdU^+^ cells among transfected cells. Scale bar = 50 μm. Abbreviations: BrdU, 5-bromo-2′-deoxyuridine; CMZ, ciliary marginal zone; CR, central retina; GR, glucocorticoid receptor; GFP, green fluorescent protein; L, lens.

We next examined whether Hes4-dependent blockade of differentiation was accompanied by prolonged proliferative capacities. We first analyzed BrdU incorporation at stage 37 following *Hes4-GR* mRNA injection. At this point of retinal histogenesis, the majority of cells in the wild-type retina are postmitotic and proliferation is restricted to the CMZ. In contrast, a dramatic increase in BrdU-positive cell number was observed in the CR of *Hes4-*overexpressing embryos (Fig. 5A, 5B).

We next assayed in lipofection experiments whether these effects on proliferation were cell autonomous (Supporting Information Fig. S6). To challenge the specificity of the *Hes4* phenotype, transfection of another *Hes* family member, *Hes2*, was performed in a parallel batch of embryos. In contrast to *Hes4*, *Hes2* is not expressed in stem cells but restricted to retinal progenitors where it acts as a gliogenic factor. We previously showed that it does so at least in part by delaying cell cycle exit of late precursor cells, while having no effect on early progenitor cell proliferation [43]. In line with this, we found that *Hes2* overexpression increased the percentage of BrdU-positive cells among transfected cells at stage 35 but not at stage 32. In contrast, proliferation rates of *Hes4*-misexpressing cells were elevated at both stages with respect to the control situation (Supporting Information Fig. S6). This suggests that both early and late retinal precursors are maintained longer in the cell cycle upon *Hes4* overexpression.

To examine the timing of cell cycle exit more directly, we performed birthdating experiments (Fig. 5C). BrdU was injected at regular intervals so that it would be constantly available from stage 34 to stage 41, and therefore mark all cells born in that period. We found that the proportion of BrdU-labeled cells among transfected cells was significantly increased following *Hes4* transfection, compared to the control situation (Fig. 5D). This clearly indicates that Hes4 delays cell cycle withdrawal of precursor cells.

Finally, we wondered whether such prolonged proliferation might account for the aforementioned imbalance between neuronal and glial cell production (Fig. 4D). We addressed this issue by counteracting *Hes4*-dependent maintenance in the cell cycle through colipofection with *Xgadd-45γ*. This cell cycle inhibitor was previously shown to accelerate retinal cell birthdate in similar assays [44]. We found that *Xgadd-45γ* indeed concomitantly rescued the *Hes4*-induced delayed cell cycle exit and the deficit in neuronal cells (Supporting Information Fig. S7), strongly suggesting a tight coupling between differentiation and proliferation defects.

### *Hes4* Misexpression Slows Down Cell Cycle Kinetics of Retinal Precursor Cells

As stated above, *Hes4* misexpression leads to a reduction of retinal size, an apparently contradictory phenotype in view of the delayed cell cycle withdrawal of retinal precursor cells. As no increase in apoptosis could be detected following *Hes4-GR* mRNA injection (data not shown), we thus wondered whether this paradox might be explained by slower cell cycle kinetics of *Hes4*-overexpressing progenitors compared to controls.

**Figure 6:**
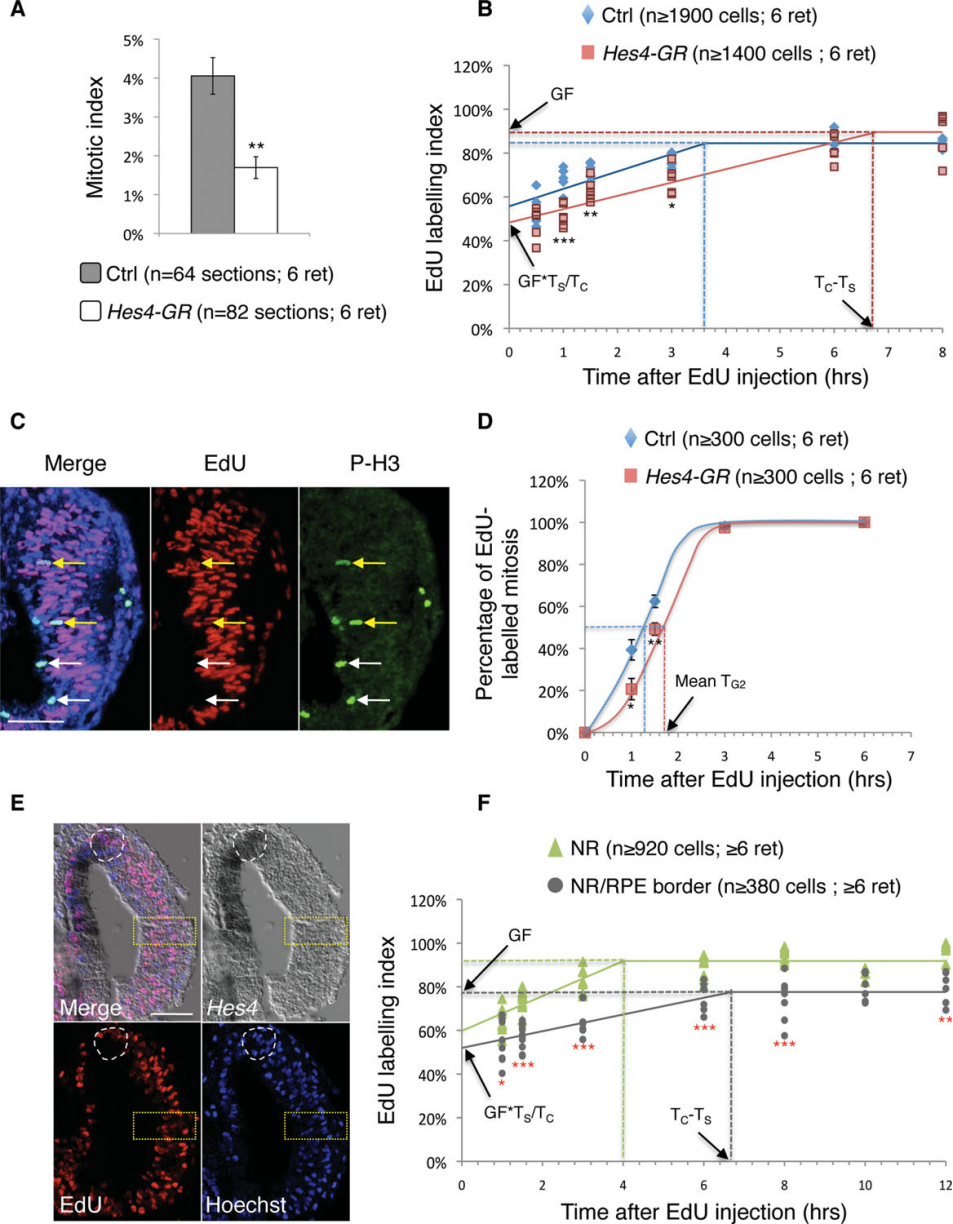
*Hes4* overexpression slows down cell cycle speed of retinal precursors. **(A):** Quantification of the mitotic index in the retinal neuroepithelium at stage 25, following *Hes4-GR* mRNA injection. **(B):** Evaluation of the EdU labeling index in the NR of control and *Hes4-GR*-injected embryos, after cumulative EdU labeling starting from stage 25. **(C, D):** G2 length evaluation *in vivo*, following *Hes4-GR* mRNA injection. (C) Typical retinal section (stage 25) stained for both phospho-Histone H3 (P-H3) and EdU, following a 1.5-hour EdU pulse. White and yellow arrows point to P-H3^+^/EdU^−^ and to P-H3^+^/EdU^+^ cells, respectively. (D) Proportion of EdU-labeled mitosis along with increasing EdU exposure times. **(E, F):** Proportion of EdU-labeled nuclei after cumulative EdU labeling, starting from stage 22. EdU detection was performed following *in situ* hybridization with a *Hes4* probe. Shown in (E) are typical retinal sections stained for both *Hes4* and EdU, following a 1-hour EdU pulse. Labeling index was measured in the *Hes4*-expressing NR/RPE border (delineated by white dotted lines in E) or in the *Hes4*-negative NR (yellow dotted lines) of control embryos. Scale bar = 50 μm. Abbreviations: EdU, 5-ethynyl-2′-deoxyuridine; GR, glucocorticoid receptor; NR, neural retina; RPE, retinal pigmented epithelium.

To address this possibility, we first evaluated the mitotic index in the retinal neuroepithelium of stage 25 embryos, using the mitotic marker phospho-Histone H3 (P-H3; [12]). P-H3 positive cell proportion was calculated among all nuclei and further corrected by the GF (percentage of proliferative cells) as determined by EdU cumulative labeling experiments (see below; Fig. 6B; Supporting Information Table 3). We found a significantly lower mitotic index in *Hes4-GR*-injected optic vesicles compared to the control (Fig. 6A), suggesting that *Hes4* overexpression slows down cell cycle speed of retinal precursors.

We next aimed at directly measuring total cell cycle (*T*_C_) and S-phase (*T*_S_) lengths in the NR through a cumulative EdU labeling assay. As shown in Figure 6B and Supporting Information Table 3, the duration of G2 + M + G1 (*T*_C_ − *T*_S_), as inferred from the time point at which the EdU labeling index reached the plateau, was longer in *Hes4*-misexpressing cells compared to wild-type ones. Calculation of *T*_S_ and *T*_C_ using the Nowakowski excel sheet [46] confirmed the hypothesis of extended cell cycle duration following *Hes4-GR* overexpression (+39% compared to the control situation; 14.73 vs. 10.62 hours) and showed a slight increase of S-phase length (+13%; 7.94 vs. 7.00 hours; Supporting Information Table 3).

Finally, in order to determine whether a specific phase of the cell cycle was particularly affected, we individually estimated G2 (*T*_G2_), M (*T*_M_), and G1 (*T*_G1_) durations. G2 length was evaluated using the percentage of labeled mitoses (PLM) paradigm [12]. Noticeably, the PLM was consistently lower in *Hes4-GR*-overexpressing retinas compared to control ones, indicating a delayed S- to M-phase progression (Fig. 6C, 6D). Mean *T*_G2_ was estimated at 1.6 hours and 1.2 hours, respectively (+33%; Fig. 6D; Supporting Information Table 3). Taking into account data gathered from the measurement of the mitotic index shown previously (Fig. 6A), we next determined *T*_M_ (see Materials and Methods section for calculation details) and finally deduced *T*_G1_ as being equal to *T*_C_ − (*T*_S_ + *T*_G2_ + *T*_M_). This revealed that increased time spent in G1 is the main parameter that accounts for the longer cell cycle observed upon *Hes4* overexpression (+148%; 4.94 vs. 1.99 hours), S and G2 phases being lengthened as well although to a lesser extent (Supporting Information Table 3).

The above data suggest that Hes4 transcription factor acts as a regulator of cell cycle speed. In line with this, the stem cell compartment of the CMZ where *Hes4* is expressed was recently shown to exhibit slow cell cycle kinetics [6]. As stated above, *Hes4* expression at the NR/RPE border of the optic vesicle likely prefigures the subpopulation of cells dedicated to form the adult RSC cohort. We thus wondered whether cells in this territory might be endowed with slow cell cycle kinetics as well. To address this issue, we performed EdU cumulative labeling in stage 22–24 wild-type embryos and analyzed EdU incorporation in two distinct territories: the *Hes4*-negative NR and the *Hes4*-expressing NR/RPE border (Fig. 6E). Calculations of *T*_S_ and *T*_C_ at the NR/RPE border yielded values of 13.57 and 20.26 hours compared to 7.47 and 11.47 hours in the NR (Fig. 6F; Supporting Information Table 3). Altogether these results strongly suggest that Hes4 contributes to the maintenance of slow cell cycle kinetics at the NR/RPE border.

## DISCUSSION

### New Insights into the Embryonic Cell-of-Origin of Adult *Xenopus* RSCs

**Figure 7:**
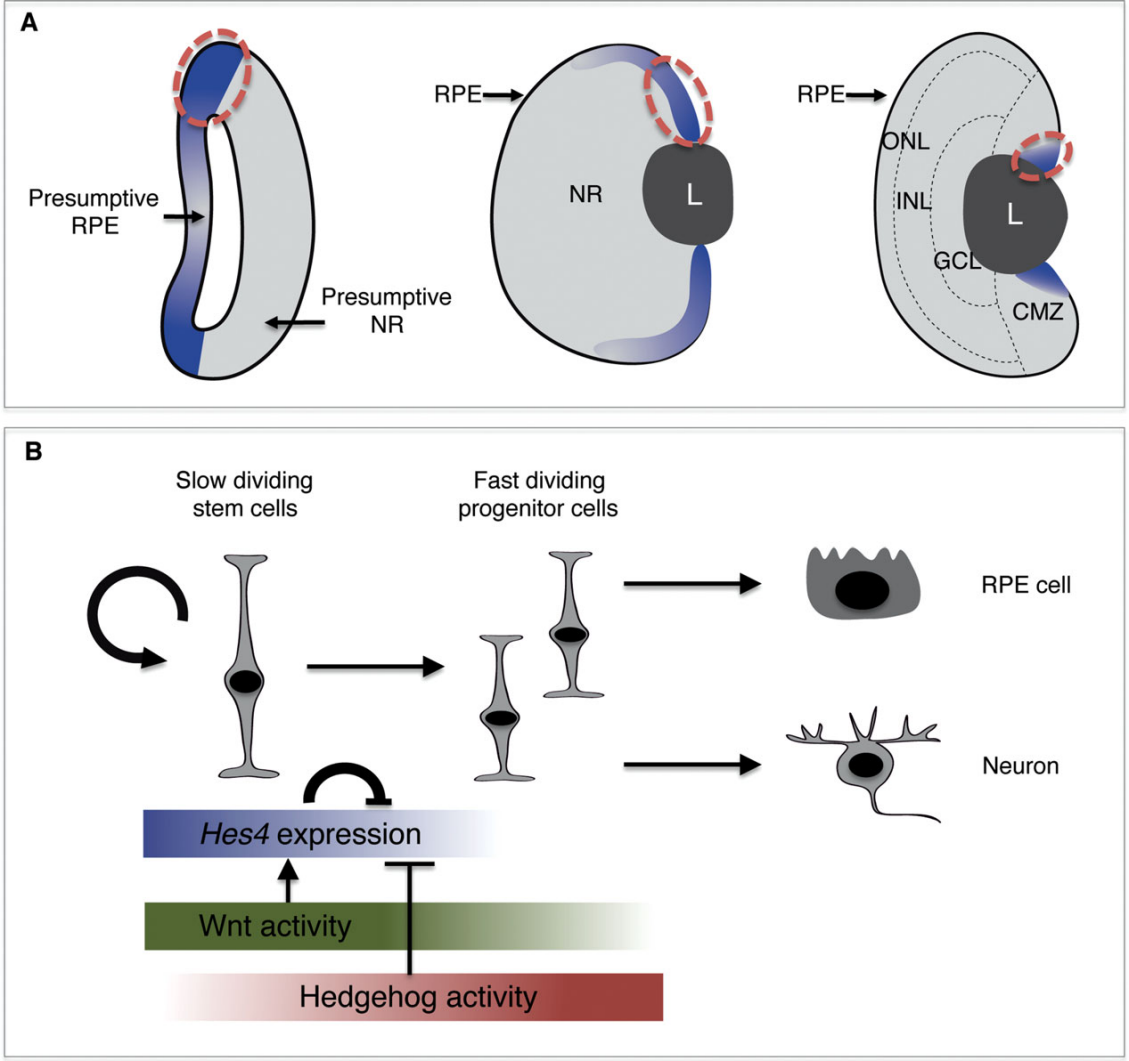
A proposed model for retinal stem cell (RSC) cell-off-origin and Hes4 function during embryonic retinogenesis. **(A):** Schema highlighting the dynamics of *Hes4* expression during retinogenesis. Adult RSCs likely originate from the most dorsal *Hes4*-expressing part of the optic vesicle (red dotted lines). **(B):** Schematic illustration of *Hes4* regulation and function in the presumptive adult RSCs. Abbreviations: CMZ, ciliary marginal zone; GCL, ganglion cell layer; INL/ONL: inner/outer nuclear layer; NR, neural retina; RPE, retinal pigmented epithelium.

Elegant tracing experiments recently allowed to identify the offspring of a single CMZ stem cell during fish postnatal life [5]. However, the embryonic lineage of adult RSCs has never been investigated so far. Our retrospective analysis of *Hes4* expression pattern suggests that these cells originate from the RPE/NR border of the optic vesicle. This hypothesis is substantiated by our finding that other stem cell-specific markers of the postembryonic CMZ display similar expression dynamic during retinogenesis: first labeling the presumptive RPE at optic vesicle stage, then the retinal margin of the optic cup, and finally the most peripheral stem-cell containing part of the CMZ in the mature retina. Importantly, it also fits with recent results from four dimensional manual cell tracking in zebrafish, suggesting that part of the optic vesicle medial layer (classically considered as prospective RPE) contributes to the CMZ [55]. Finally, such an early restricted expression of RSC markers challenges the classical schematic representation of retinal histogenesis, which implies that RSCs are initially found throughout the NR of the newly emerged optic vesicle and thereafter persist in its periphery as central cells differentiate. It rather suggests that a presumptive RSC cohort is already segregated from both the NR and RPE lineages as early as the optic vesicle stage (Fig. 7A).

### Hes4 Maintains Presumptive RSCs in a Proliferative and Undifferentiated State

Our gain of function analysis revealed that *Hes4* misexpression prevents retinal precursors from committing toward retinal fates. Of note, Hes4 ability to block neurogenesis is a function shared by many other Hes family members that are known to transcriptionally repress or functionally antagonize several proneural factors [24]. In particular, cHairy1 has proved to be required to prevent CMZ cells from differentiating into neurons and to be sufficient to endow progenitor cells of the CR with CMZ-like cell properties [38]. We found here that the role of Hes4 during *Xenopus* retinal development may also extend to an inhibition of the RPE differentiation program. Besides, our results indicate that the Hes4-dependent blockade of neuronal and RPE determination is accompanied by maintenance in a proliferative state. Whether the former effect is a consequence of the latter or *vice versa* remains to be determined. However, both appear tightly coupled since *Hes4*-induced defective neurogenesis, as observed in lipofection experiments, can be rescued by forcing cells to exit the cell cycle. We thus conclude that the *Hes4* phenotype observed in our overexpression experiments reflects a function dedicated to maintain presumptive adult RSCs in an undifferentiated and proliferative state towards adulthood.

### Hes4 Regulates Cell Cycle Kinetics

Our study highlights that Hes4 might also constitute a crucial regulator of cell cycle kinetics. Cell cycle speed is known to vary during the time course of vertebrate retinogenesis [44, 56, 57], a phenomenon that has been associated with the transition from proliferative to neurogenic divisions in other parts of the central nervous system [58]. However, comparative analysis of proliferation kinetics between different retinal compartments at a given stage has not been performed so far. We found in this study that *Hes4*-expressing cells at the NR/RPE border of the optic vesicle exhibit a much longer cell cycle compared to NR precursors. This likely constitutes a hallmark of neural stem cells as observed in adult RSCs of the CMZ ([6] and our own unpublished data). How cell cycle speed is modulated in these cells is poorly documented. We here demonstrated that Hes4 prolongs cell cycle duration, mainly through a lengthening of G1 phase. In line with this result, sustained overexpression of *Hes1* in brain neural progenitors was previously shown to downregulate expression of cell cycle regulators such as *CyclinD1* and *CyclinE2* and to cause G1 phase retardation [26]. Our data thus strongly support the hypothesis that Hes4 regulates some aspects of RSC homeostasis through the maintenance of slow cell cycle kinetics.

### *Hes4* Is Tightly Regulated by Multiple Inputs

We recently discovered that opposed and counterbalancing functions of Wnt and Hedgehog signaling modulate neural stem/progenitor cell proliferation in the postembryonic CMZ and that the two pathways reciprocally regulate each other's activity [41]. In line with this model, we found that the two pathways exhibit opposite effects on *Hes4* expression in the forming CMZ.

The Hes4-dependent maintenance in the cell cycle associated with slow cell cycle kinetics is highly reminiscent of the phenotype resulting from Hedgehog signaling blockade in the retina [12]. Consistent with this, but in sharp contrast with *Hes1* regulation in the mouse retina [53], we found that the Hedgehog pathway negatively impacts on *Hes4* expression in the forming CMZ. In line with our previously proposed model [12, 59], it would thus be interesting to investigate whether Hedgehog-driven downregulation of *Hes4* is required for the transition from a slow cycling RSC phenotype to a fast cycling progenitor state (Fig. 7B).

Our results also highlight that the canonical Wnt pathway (a) is active in the presumptive RSC subpopulation and (b) positively modulates *Hes4* levels in the forming CMZ. These results suggest that Wnt signaling may function upstream *Hes4* in the maintenance of RSCs in a proliferative and undifferentiated state during retinogenesis (Fig. 7B). This hypothesis is substantiated by the fact that the Wnt pathway is required for proliferation in the postembryonic *Xenopus* CMZ [10], and that Wnt-dependent prevention of chick CMZ cell differentiation is mediated by cHairy1 [38]. A positive link between the Wnt receptors frizzled 5/8 and *Hes1* gene has also recently been observed in the context of cell proliferation in the mouse neuroretina [60]. Besides, activation of the Wnt pathway through overexpression of stabilized β-catenin in mouse [61] or in the zebrafish *apc* mutant [62] results in lower proliferation rates in the peripheral retina. It is therefore tempting to speculate that Wnt signaling might control as well Hes4-driven slow cell cycle speed of neural stem cells in the *Xenopus* retina.

In addition to these opposite influences of Wnt and Hedgehog signaling, we found that Hes4 is able to repress its own transcription. Such a negative feedback loop has previously been reported for Hes1 and is known to contribute to oscillations of its expression in brain neural precursors [26, 63]. However, *Hes4*-expressing cells in the retina exhibit features that are strikingly reminiscent of boundary cell properties (constitutive repression of proneural genes and reduced proliferation rates). In these boundary cells *Hes1*, like other *Hes*-related genes, does not oscillate and is rather persistently expressed at high levels [20, 27]. This sustained upregulation is due to the action of Id factors that prevent Hes1 from binding to its own promoter [64]. As shown here, *Id2* and *Hes4* exhibit a similar expression pattern in the developing retina. It would thus be interesting to determine whether a similar interaction leading to sustained *Hes4* expression might be at work in RSCs.

## CONCLUSION

Altogether, our study opens new avenue onto the cell-of-origin of adult RSCs and the involvement of the transcriptional repressor Hes4 in their maintenance during embryonic development. Our results indeed suggest that *Hes4* expression at the NR/RPE border of the optic vesicle foreshadows the adult RSC pool of the CMZ. Besides, our functional analysis converges toward a model whereby Hes4 acts as a safeguard of neural stemness features in this cell subpopulation by preventing its differentiation and maintaining it in a slowly proliferative state.

## Supplementary material

Figure S1. Hes4 expression on whole mount embryos. Whole mount in situ hybridization analysis of Hes4 expression (lateral views of tailbud heads, anterior to the right). In addition to the eye, Hes4 expression is detected in several parts of the brain and spinal cord, in the branchial arches (white arrow in B-E) and in the pronephros (black arrowhead in B-D). For more details on Hes4 expression pattern in whole mount embryos, see [65]. Scale Bar: 300 μm.

Figure S2. Expression pattern of the retinal stem cell markers Id2 and Wnt8b during retinogenesis. In situ hybridization analysis of Id2 and Wnt8b expression during retinal development. Following expression in the presumptive RPE at the optic vesicle stage (stage 24), both genes get restricted to the peripheral RPE and forming CMZ in the optic cup (stage 32). Their expression in the CMZ of the mature retina (stage 41) is confined to the most peripheral region where neural stem cells reside (see magnifications of the dorsal CMZ on the right panels). Note that both Id2 and Wnt8b exhibit a more intense staining dorsally than ventrally. Scale Bar: 50 μm.

Figure S3. Hes4 is expressed in domains exhibiting high Wnt and low Hedgehog activity. Comparative in situ hybridization analysis of Hes4 expression profile with that of eGFP (in Wnt-responsive transgenic embryos) and Gli1 (as a readout of hedgehog signalling activity). Black and red dotted lines respectively delineate the RPE/NR border and the stem cell zone of the CMZ. Red and black arrows respectively point to the differentiating central RPE and the retinal margin. Drawings on the right schematize Hes4 expression and domains with Wnt or Hedgehog activity at stage 25 and 35. Scale Bar: 50 μm. Hes4 and retinal stemness

Figure S4. Hes4 represses its own transcription. (A) Whole mount in situ hybridization analysis of N-Tubulin and Hes4 expression on stage 23 embryos injected with Hes4VP16-GR mRNA. Arrows indicate ectopic expression in the epidermis (anterior on the right, dorsal side up). Of note, a similar result was obtained with a probe designed in the 3′UTR of the Hes4 gene, confirming that it was not due to crosshybridization with the Hes4VP16-GR mRNA. (B) Whole mount in situ hybridization analysis of Rx and Hes4 expression on stage 20 embryos, following injection of control or Hes4 morpholinos (Mo). Note the reduction of Rx staining in the eye field (black arrow) and the increase of Hes4 expression (white arrows) on the injected side (Inj). N indicates the proportion of embryos exhibiting the corresponding phenotype. Scale bar: 500 μm (A) or 300 μm (B).

Figure S5. Hes4 expression is insensitive to a 24-hour Notch signalling blockade. (A-E) In situ hybridization analysis following a 24- (from stage 25 to stage 35; A, B) or a 48- hour (from stage 25 to stage 39; C, D) treatment with the Notch inhibitor DAPT. (A, C) Expression of the Notch target gene HRT1 (lateral views of the head) serves as a control of DAPT efficiency [42]. Arrows point to the brain labelling, which is clearly decreased upon DAPT treatment. (B, D) Typical Hes4 staining on retinal sections from control or treated embryos as indicated. Shown on the right are higher magnifications of the dorsal CMZ, delineated with dotted lines. (E) Quantification of Hes4 staining area in the dorsal CMZ in each condition. (F) qPCR analysis of retinal Hes4 expression at stage 35 following a 24-hour DAPT treatment. N-Tubulin serves as a control of drug efficiency. Scale bar: 50 μm.

Figure S6. Hes4 overexpression prolongs proliferation of retinal progenitors. (A) Typical sections of stage 32 retinas lipofected with GFP and immunostained for both GFP and BrdU. White and yellow arrows point to a GFP+/BrdU- and to a GFP+/BrdU+ cell, Hes4 and retinal stemness respectively. (B) Quantification of BrdU incorporation (3-hour pulse) in retinal clones at stage 32 or 35, following Hes4 or Hes2 lipofection. Scale Bar: 50 μm.

Figure S7. Hes4-dependent reduction of neurogenesis can be rescued by forcing cell cycle exit. (A, B) Analysis of cell type distribution in stage 41 retinas, following Hes4, Xgadd-45γ or Hes4 plus Xgadd-45γ lipofection. (A) Typical sections of retinas transfected with the indicated construct. (B) Quantification of neurons and glia among transfected cells in each condition. (C) Birthdating experiments in retinas lipofected with Hes4, Xgadd-45γ or Hes4 plus Xgadd-45γ. GCL: ganglion cell layer; INL/ONL: inner/outer nuclear layer. Scale Bar: 25 μm.
