# Supplementary figures and images for: Hes4 Controls Proliferative Properties of Neural Stem Cells During Retinal Ontogenesis
Source: Stem Cells. 2012 Sep 11;30(12):2784–95. doi: 10.1002/stem.1231 (PMC3549485; doi:10.1002/stem.1231)

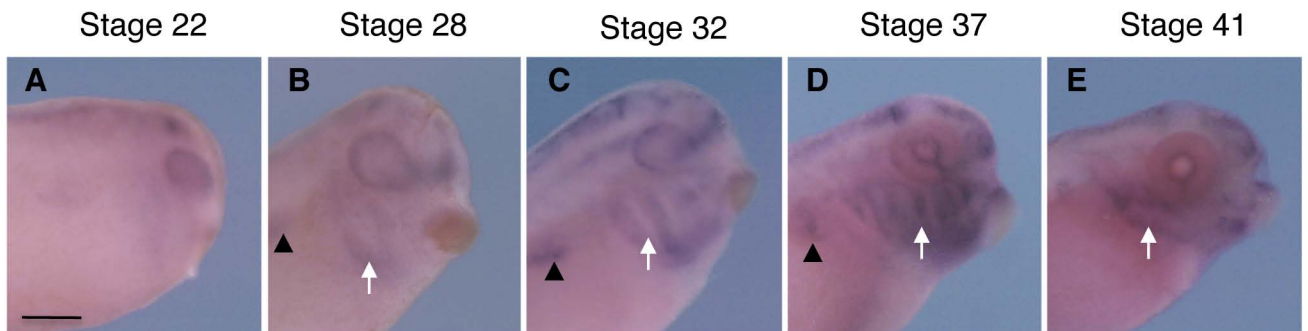

Supplement: Supplementary file 1 [file stem0030-2784-SD1.pdf]

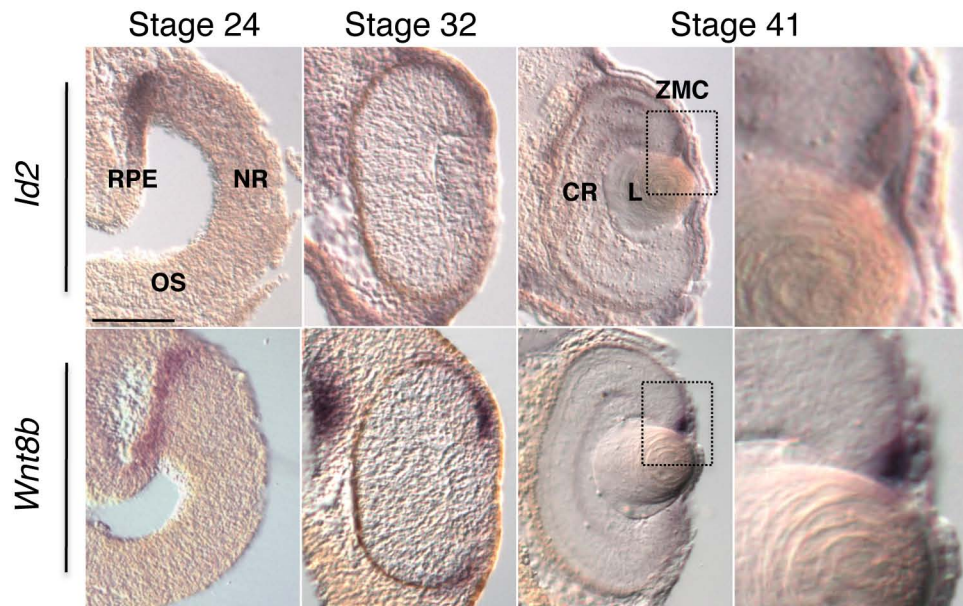

Supplement: Supplementary file 2 [file stem0030-2784-SD2.pdf]

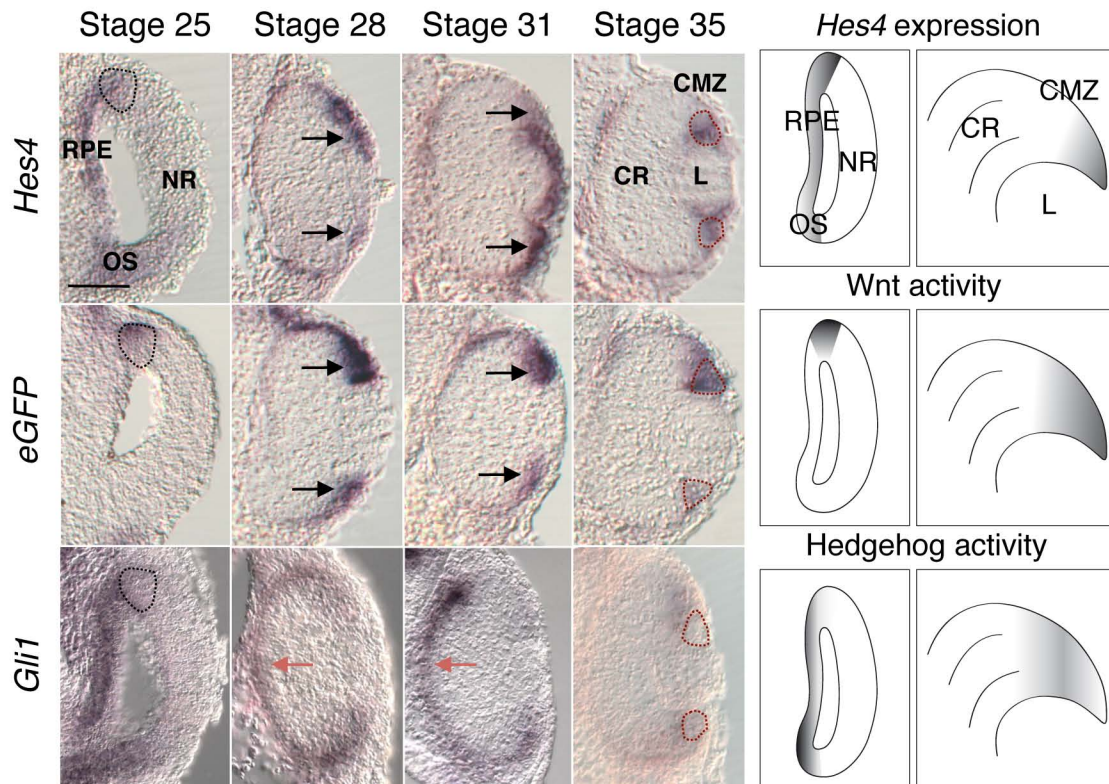

Supplement: Supplementary file 3 [file stem0030-2784-SD3.pdf]

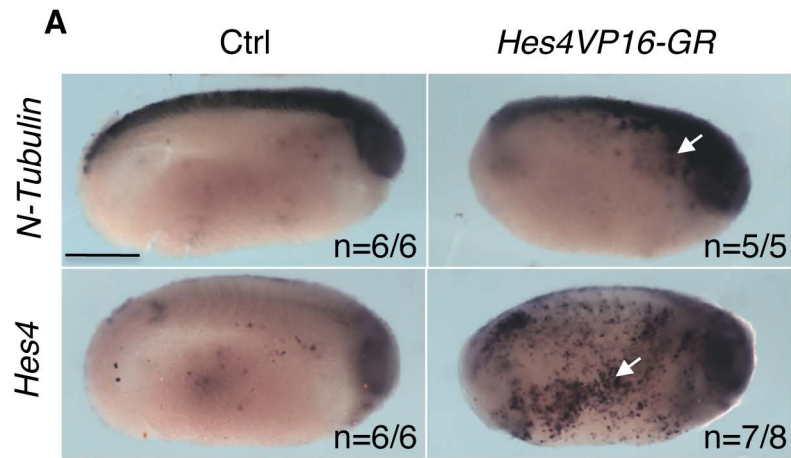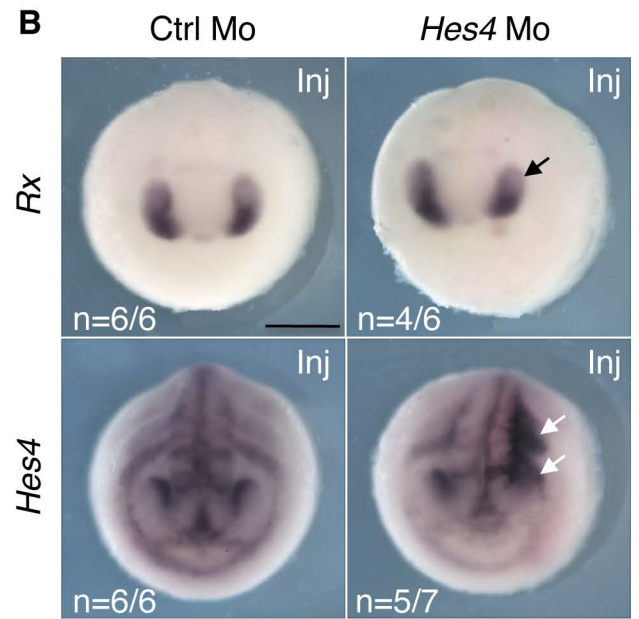

Supplement: Supplementary file 4 [file stem0030-2784-SD4.pdf]

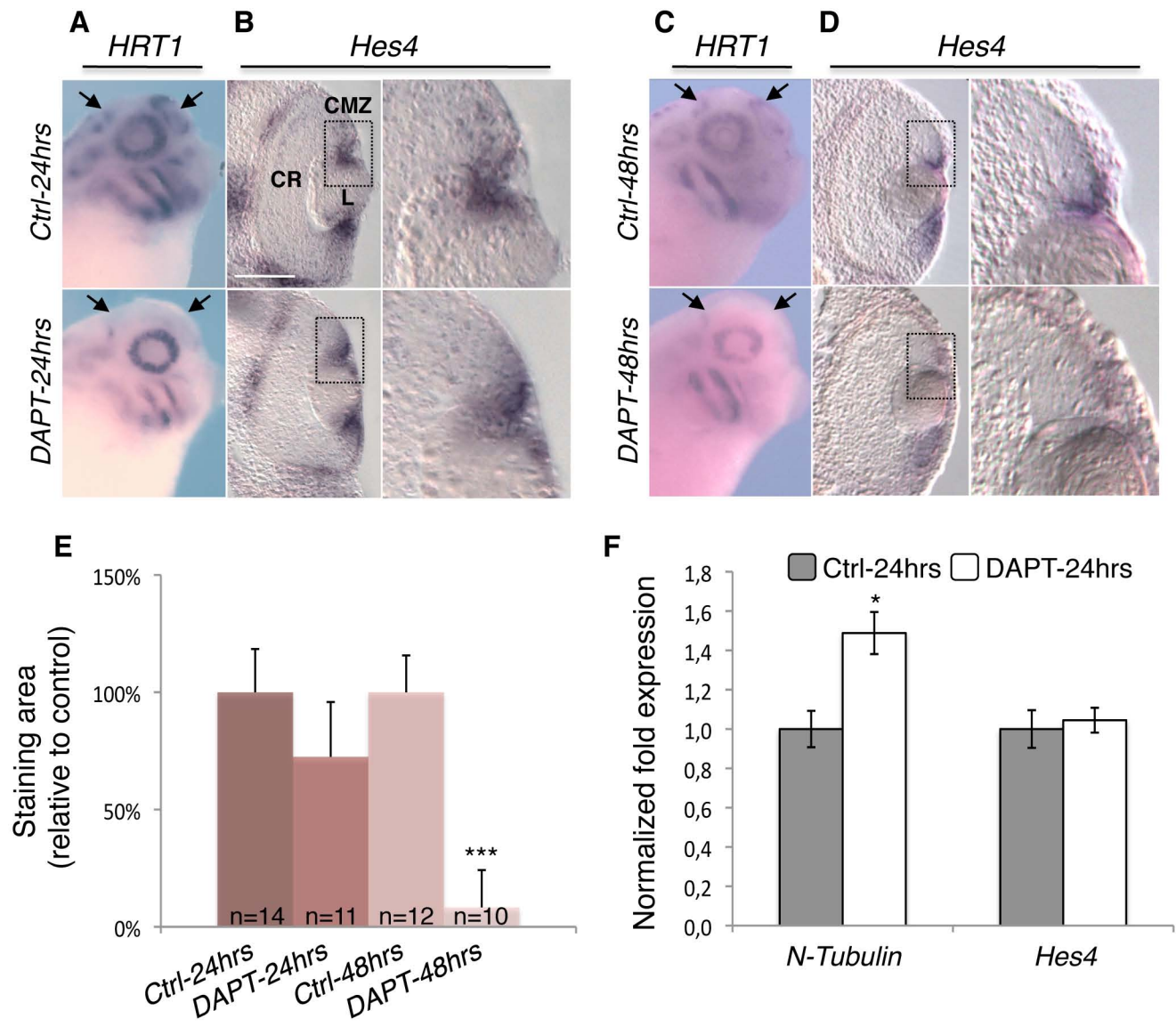

Supplement: Supplementary file 5 [file stem0030-2784-SD5.pdf]

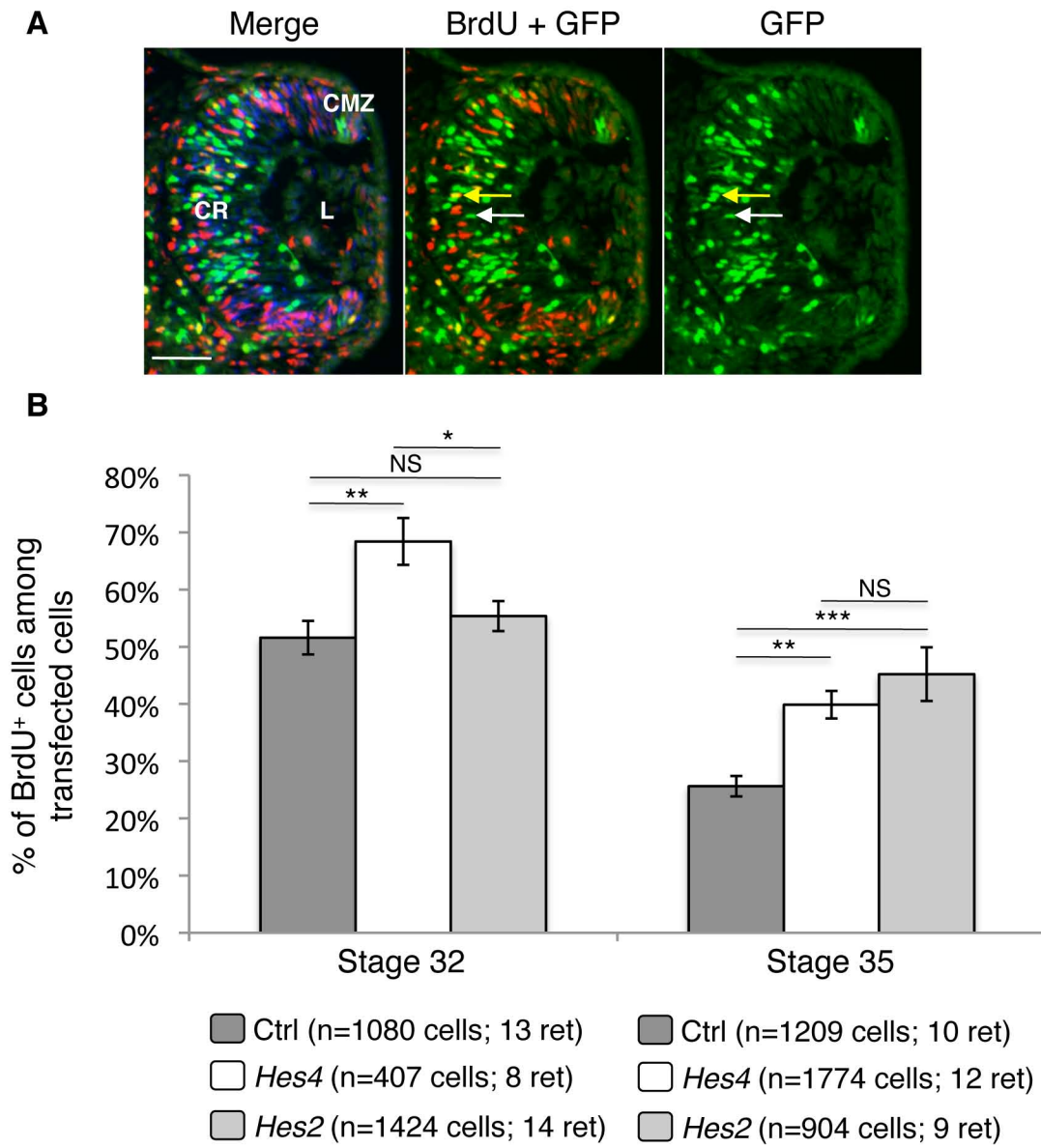

Supplement: Supplementary file 6 [file stem0030-2784-SD6.pdf]

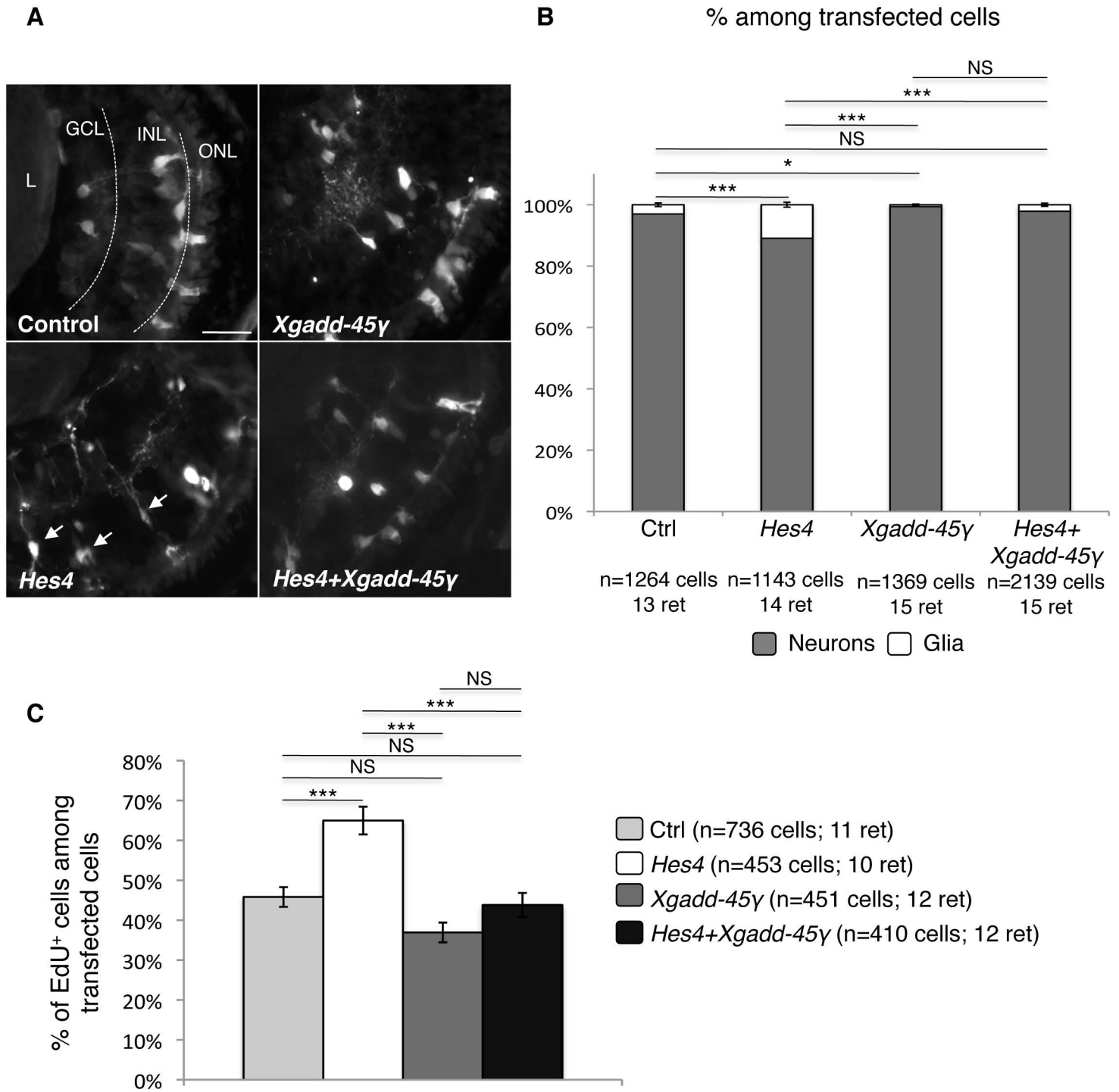

Supplement: Supplementary file 7 [file stem0030-2784-SD7.pdf]
