# Supplementary material for: Hes4 Controls Proliferative Properties of Neural Stem Cells During Retinal Ontogenesis
Source: Stem Cells. 2012 Sep 11;30(12):2784–95. doi: 10.1002/stem.1231 (PMC3549485; doi:10.1002/stem.1231)
Supplement: Supplementary file 8 [file stem0030-2784-SD8.pdf]

Supplementary Table 1. List of antibodies used.

Antibody	Dilution	Manufacturer
Mouse monoclonal anti-BrdU	1:100	Becton Dickinson
Rabbit polyclonal or mouse monoclonal anti-GFP	1:400	Molecular Probes
Chicken polyclonal anti-GFP (*)	1:500	Aves Labs
Rabbit polyclonal anti-phospho-Histone H3	1:500	Upstate biotechnology
Rabbit polyclonal anti-active caspase 3	1:500	BD Pharmingen
Mouse monoclonal anti-syntaxin	1:500	Sigma
Mouse monoclonal anti-RPE XAR1	1:10	A gift from Don Sakaguchi
Mouse monoclonal anti-calbindin	1:100	Swant
Anti-mouse or anti-rabbit fluorescent secondary antibodies (Alexa 488 or 594)	1:1000	Molecular Probes

(*) Antibody used on paraffin-sections following a 10 mn antigen unmasking treatment in boiling Heat-mediated Antigen Retrieval Buffer (10mM sodium citrate, 0.05% Tween 20, pH 6.0)
