# Supplementary material for: Hes4 Controls Proliferative Properties of Neural Stem Cells During Retinal Ontogenesis
Source: Stem Cells. 2012 Sep 11;30(12):2784–95. doi: 10.1002/stem.1231 (PMC3549485; doi:10.1002/stem.1231)
Supplement: Supplementary file 9 [file stem0030-2784-SD9.pdf]

Supplementary Table 2. List of primers used in qPCR experiments.

***Xenopus laevis* qPCR primers**

gene	FW primer	RV primer
Hes4	CCCCCTCCAGCCAACAACCA	GGGGGAGATGGCCTCTGCTG
Patched1	CAGCTGCCCAGCCGAGGGTA	GGGCGAAATTGGCATCGCAGTA
CyclinD1	CATCCGCAAACACGCCCAGA	GACACTGCCAGCGGCGATCA
N-Tubulin	CCCGTGCCATCCTTGTGGATTT	GCCCAGTTATTGCCAGCACCACTT
ODC (reference gene)	GCTTCTGGAGCGGGCAAAGGA	CCAAGCTCAGCCCCCATGTCA
RPL8 (reference gene)	CCACGTGTCCGTGGTGTGGCTA	GCGCAGACGACCAGTACGACGA
