# Supplementary material for: Hes4 Controls Proliferative Properties of Neural Stem Cells During Retinal Ontogenesis
Source: Stem Cells. 2012 Sep 11;30(12):2784–95. doi: 10.1002/stem.1231 (PMC3549485; doi:10.1002/stem.1231)
Supplement: Supplementary file 10 [file stem0030-2784-SD10.pdf]

Supplementary Table 3. Labelling index and cell cycle parameters in the NR and NR/RPE border.

A

		EdU exposure (hrs)							
		0.5	1	1.5	3	6	8	10	12
**NR Ctrl Stage 25**	LI (% ± s.e.m.)	53.80 ± 2.77	67.89 ± 2.04	71.79 ± 1.48	77.06 ± 1.24	85.04 ± 1.80	83.92 ± 0.82	ND	ND
	Total cell count	1927	2108	1785	3008	1948	2470		
**NR *Hes4-GR* Stage 25**	LI (% ± s.e.m.)	48.65 ± 2.84	51.45 ± 2.04	62.70 ± 1.95	68.52 ± 2.44	83.24 ± 2.52	89.60 ± 4.18	ND	ND
	Total cell count	1394	2153	2076	2181	2457	2374		
**NR Stage 22**	LI (% ± s.e.m.)	ND	65.60 ± 1.96	73.93 ± 1.16	82.95 ± 1.87	89.11 ± 1.93	94.81 ± 1.10	86.04 ± 1.23	97.03 ± 1.45
	Total cell count		1008	1318	983	1698	1043	1361	927
**NR/RPE border Stage 22**	LI (% ± s.e.m.)	ND	55.84 ± 3.25	57.98 ± 1.64	62.84 ± 1.95	75.17 ± 2.16	74.67 ± 3.40	79.51 ± 2.55	79.62 ± 2.97
	Total cell count		644	1057	724	853	387	559	493

B

	Cell-cycle parameters							
	**T_C-T_S**	**GF*T_S/T_C**	**GF**	**T_C**	**T_S**	**T_{G2}**	**T_M**	**T_{G1}**
**NR Ctrl Stage 25**	3.62 hrs	0.56	84.48%	10.62 hrs	7.00 hrs	1.20 hrs	0.43 hrs	1.99 hrs
**NR *Hes4-GR* Stage 25**	6.79 hrs	0.48	89.60%	14.73 hrs	7.94 hrs	1.60 hrs	0.25 hrs	4.94 hrs
**NR Stage 22**	4.00 hrs	0.60	91.78%	11.47 hrs	7.47 hrs	ND	ND	ND
**NR/RPE border Stage 22**	6.69 hrs	0.52	77.61%	20.26 hrs	13.57 hrs	ND	ND	ND
